# Task‐dependent spatial processing in the visual cortex

**DOI:** 10.1002/hbm.26489

**Published:** 2023-10-09

**Authors:** G. Bertonati, M. B. Amadeo, C. Campus, M. Gori

**Affiliations:** ^1^ Unit for Visually Impaired People (U‐VIP) Istituto Italiano di Tecnologia Genoa Italy; ^2^ Department of Informatics, Bioengineering, Robotics and Systems Engineering (DIBRIS) Università degli Studi di Genova Genoa Italy

**Keywords:** EEG, space perception, visual brain

## Abstract

To solve spatial tasks, the human brain asks for support from the visual cortices. Nonetheless, representing spatial information is not fixed but depends on the reference frames in which the spatial inputs are involved. The present study investigates how the kind of spatial representations influences the recruitment of visual areas during multisensory spatial tasks. Our study tested participants in an electroencephalography experiment involving two audio–visual (AV) spatial tasks: a spatial bisection, in which participants estimated the relative position in space of an AV stimulus in relation to the position of two other stimuli, and a spatial localization, in which participants localized one AV stimulus in relation to themselves. Results revealed that spatial tasks specifically modulated the occipital event‐related potentials (ERPs) after the onset of the stimuli. We observed a greater contralateral early occipital component (50–90 ms) when participants solved the spatial bisection, and a more robust later occipital response (110–160 ms) when they processed the spatial localization. This observation suggests that different spatial representations elicited by multisensory stimuli are sustained by separate neurophysiological mechanisms.

## Graphical abstract

The kind of spatial representations may modulate visual cortical activity. Participants perform audio–visual spatial bisection and localization during electroencephalography. Stronger early contralateral occipital activation (50‐90 ms) during spatial bisection and greater later occipital recruitment (110‐160 ms) during spatial localization were observed. These results suggest that different spatial tasks are sustained by separate neurophysiological mechanisms.

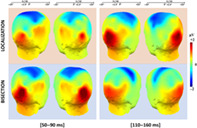

## 1 INTRODUCTION

Visual modality is the main sense resolving difficult tasks such as spatial representation. Vision dominates space perception, enabling the simultaneous acquisition of environmental proprieties. It extracts the most detailed information about the surroundings and it shapes and biases other sensory modalities in creating an integrated spatial percept (Thinus‐Blanc & Gaunet, 1997; Tinti et al., 2006). Indeed, to build a coherent representation of the world, infants combine spatial information originating from different senses (Bremner et al., 2008) and learn to use the visual system as the main background for the spatial alignment of the other sensory modalities (King, 2009, 2014). In this regard, children show strong visual dominance in audio–visual (AV) space perception (Gori et al., 2012).

The strong connection of the visual modality with space perception suggests that recruiting visual cortices might be essential for building spatial representations through different sensory modalities. Notably, past research showed that the visual areas can be recruited by the processing of multisensory stimuli, which are involved in different experimental tasks (Alais et al., 2010; Murray, Thelen, et al., 2016), such as detection and identification tasks (Fort, Delpuech, Pernier, & Giard, 2002; Fort, Delpuech, Pernier, Giard, & Thomas, 2002; Giard & Peronnet, 1999; Molholm et al., 2002; Raij et al., 2010; Talsma & Woldorff, 2005; reviewed in De Meo et al., 2015), and they are specifically recruited in the processing of spatial information. For example, the ventriloquist effect, where vision dominates the localization of incongruent but reliable AV stimuli, activates the occipital regions (Busse et al., 2005) and evokes a neural process over the posterior scalp that depends on the spatial congruity of the audio and visual sources (Gondan et al., 2005; Teder‐Sälejärvi et al., 2005). Regarding the auditory modality, other studies demonstrated that lateralized task‐irrelevant sounds elicit a contralateral event‐related potential (ERP) over the occipital scalp, appearing between 200 and 450 ms after the sound onset (the auditory‐evoked contralateral occipital positivity [ACOP]; Matusz et al., 2016; McDonald et al., 2013). This cross‐modal activation of the extrastriate visual cortex by the spatialized sound facilitates discrimination of a subsequent visual target at the location of the sound (Feng et al., 2014). A more recent study showed that the ACOP is elicited only when discrimination of the sound location is required, and not with the judgment of sound's non‐spatial features. This suggests that cross‐modal activation of the visual cortices depends on the spatial dimension of the stimulus (Retsa et al., 2020). Finally, tactile spatialized stimulations were found to elicit activation of the visual cortex, such as in the tactile discrimination of stimuli orientation and in the perception of tactile motion (Hagen et al., 2002; Sathian et al., 2004; Zangaladze et al., 1999). An interesting point of discussion is the recruitment of visual areas by spatial tasks of a more complex nature, such as those requiring the construction of a metric representation (i.e., a layout that requires the estimation and comparison of different locations in space). In this regard, sighted adults showed an early response of areas likely involving the visual cortex during an auditory spatial bisection task (Campus et al., 2017). In this task, the experimenters asked participants to listen to three consecutive sounds and to identify whether the second sound was spatially closer to the first or the third sound. To do so, listeners had to build a spatial metric representation of the sounds as, first, they had to localize the acoustic sources in the space and then connect the spatial locations to each other. The construction of a metric spatial representation evoked a response in a time window between 50 and 90 ms from the second stimulus onset that was specific to the occipital areas. Some have hypothesized that recruitment of the occipital regions by the spatial bisection task is linked to the high spatial resolution and flexibility that only the visual cortices can achieve (Gori et al., 2020a). Finally, a recent study showed that the spatial‐specificity of visual areas persists in response to multisensory spatial inputs (Gori et al., 2023).

Overall, these findings provided evidence of a cortical system of space perception that is recruited by different sensory modalities during various kinds of spatial representation. Although the present study does not target sensory deprivation, further knowledge about spatial circuits derives from the study of blindness as a model to investigate spatial processing (and the related cortical activity). Past studies have indicated that blind individuals could localize acoustic sources in all areas of space (Battal et al., 2020), with enhanced abilities in the peripheral locations compared to the central ones (Fieger et al., 2006; Röder et al., 1999), and with evidence of cross‐modal plasticity in the visual cortex (Collignon et al., 2009; Gougoux et al., 2005; Renier et al., 2010; reviewed in Röder et al., 2022). In addition, a recent study revealed that, in visually impaired infants, auditory localization abilities are comparable to those of sighted children (Gori et al., 2021). Finally, the ACOP was more pronounced in blind people compared to sighted individuals, suggesting that this contralateral positive potential is not intrinsically visual in nature (Amadeo, Störmer, et al., 2019). Despite this evidence, other studies reported that visual impairment negatively affects blind individuals in performing more elaborate auditory and tactile spatial tasks (Bertonati et al., 2020; Gori et al., 2014; Setti et al., 2018, 2022). For example, congenitally blind people showed strong deficits in performing an acoustic spatial bisection task, but not a minimal audible angle task (Gori et al., 2014), associated with a reduced occipital response (Amadeo, Störmer, et al., 2019; Campus et al., 2019; Gori et al., 2020a; Tonelli et al., 2020). Overall, findings on the spatial abilities and correlates of visually impaired individuals may seem contradictory as they imply, on the one hand, that lack of vision does not significantly affect the development of some spatial skills (Battal et al., 2020; Fieger et al., 2006; Röder et al., 1999), but, on the other hand, that the visual experience is crucial in shaping finer spatial abilities in the auditory and tactile modalities (Bertonati et al., 2020; Gori et al., 2014). Nonetheless, these controversial results may also simply indicate that the effect of visual deprivation on the space perception is not uniform but depends on the task and the context to which the spatial information belongs. This observation is supported by the cross‐sensory calibration theory (Gori, 2015), which assumes that during childhood vision calibrates audition and touch for some spatial skills, but not for others. In particular, vision plays a crucial role in shaping complex spatial information (such as the spatial bisection; Campus et al., 2019; Gori et al., 2020b) but is less fundamental for encoding other spatial configurations (such as the spatial localization; Röder et al., 1999; Rohlf et al., 2020).

Based on all these considerations in sighted and blind individuals, we hypothesize that spatial configurations rely on different neural activations. Specifically, an early occipital activation may be more present during a spatial metric representation than a simpler localization task. Indeed, this component has been previously observed in sighted people during auditory spatial bisection, for which visual calibration is necessary (Campus et al., 2017; Gori et al., 2023), but not in blind people who struggle with it (Amadeo, Campus, et al., 2019; Campus et al., 2019; Gori et al., 2020b). Conversely, the spatial localization, which is independent of visual calibration (Rohlf et al., 2020) and related to higher performance and stronger cortical activity in the blind population (Fieger et al., 2006; Röder et al., 1999), may rely more on later occipital recruitment, previously associated with spatial attention processing (Di Russo et al., 2002).

Thus, in the present study we tested whether the brain processes the space domain differently according to the type of spatial representation that the stimuli elicit. To investigate this aspect, we tested healthy participants in an electrophysiological study involving AV spatial bisection and localization tasks. On the one hand, we chose these tasks because they elicit different ways of spatially representing stimuli: in spatial localization, positions are defined by coordinates of a single point, while in spatial bisection positions are relative and require the construction of a spatial metric among multiple stimuli. On the other hand, we decided to use bimodal (rather than unisensory) stimuli to likely elicit neural activation of the visual and auditory cortices and, consequentially, check that the task‐dependent neural modulation was specific to the visual and not auditory areas. Thus, we compared the neural response during the two different AV tasks to explore cortical modulation elicited by different spatial processing.

## 2 MATERIALS AND METHODS

### 2.1 Participants

We recruited 17 adults to participate in the study (10 females, mean age ± standard deviation [SD]: 24 ± 3.08 years old). We decided sample size based on previously published studies testing the neural correlates of spatial abilities of healthy and visually impaired adults during similar tasks (Amadeo, Campus, et al., 2019; Campus et al., 2017, 2019; Gori et al., 2023). As in these studies results with large effect size were reported, we performed a priori power analysis with estimated effect size Cohen's *d* = 0.80 (two‐tailed *t*‐test, *α* = .05), that determined a minimum sample size of 15 participants. All participants reported they had no history of neurological, cognitive, or sensory deficits and they gave written informed consent before testing. The ethics committee of the local health service (Comitato etico, ASL 3 Genova) approved the study, which was conducted in line with the Declaration of Helsinki.

### 2.2 Setup, stimuli, and procedure

Participants sat at a distance of 180 cm from the center of an array of 23 speakers/light emitting diodes (LEDs) spanning ±25° of visual angle (0° represented the central speaker/LED, with negative values on the left and positive values on the right). Each speaker was spatially aligned to one LED so that participants perceived sounds and flashes as originating from the same source.

**FIGURE 1:**
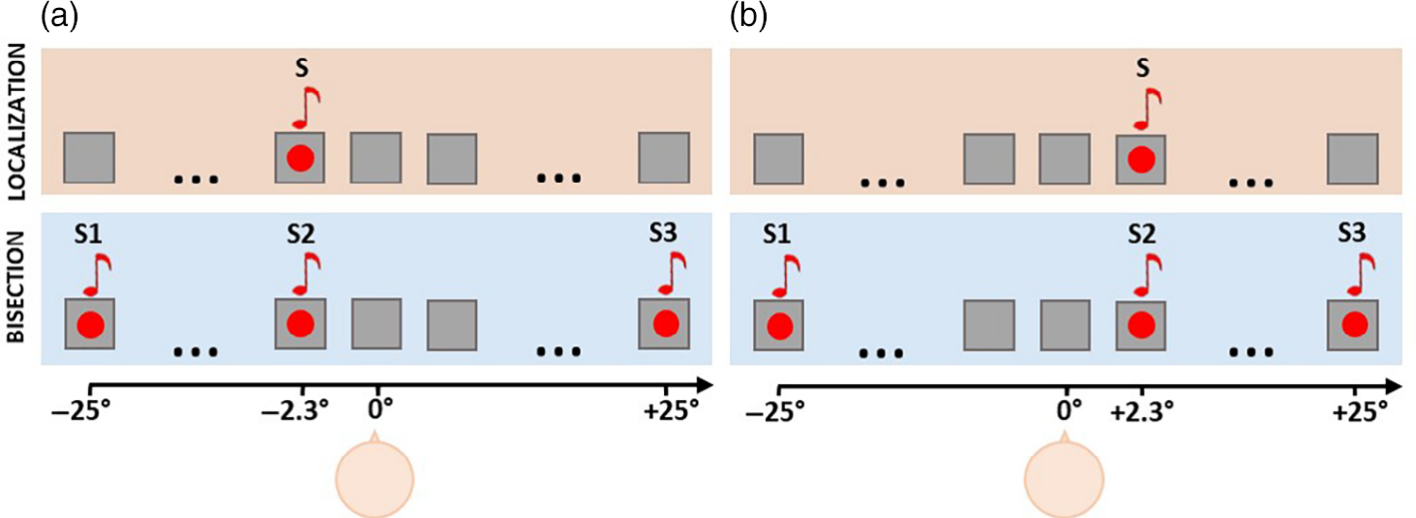
Two experimental conditions according to S2/S positions in space, for the spatial localization task (top row) and the spatial bisection task (bottom row): (a) S2 and S from −2.3°, (b) S2 and S from +2.3°. In the bisection task, S1 and S3 were always delivered at −25° and +25°, respectively.

The setup reproduced AV stimuli, each consisting of a single sound (60 dB sound pressure level (SPL) at ears' level, 500 Hz) concurrently presented with a single red flash (2.3° diameter, 20 cd/m^2^ luminance). Participants performed a spatial bisection task and a spatial localization task (Figure 1). In the spatial bisection task, each trial consisted of three AV stimuli (namely S1, S2, and S3; duration 75 ms each) played at three different spatial positions and time lags. S1 and S3 were always played at −25° and +25°, respectively, and separated by a fixed time interval of 1.5 s. S2 could be presented randomly from either −2.3° or + 2.3° in space, and at either −250 ms or +250 ms in time (with 0 ms representing the middle of the 1.5 s temporal sequence). The time separation between S1, S2, and S3 was sufficiently large to ensure a complete decay of the ERP response between the stimuli. In each trial, participants evaluated whether S2 was spatially farther from S1 or S3. In the spatial localization task, a trial consisted of a unique AV stimulus (S) reproduced from either −2.3° or +2.3°. Participants localized S by identifying its position as being more on the left (−2.3°) or on the right (+2.3°) than the center of the array (0°, aligned with the subject body midline). For both tasks, subjects provided their answers after the stimuli presentation by pressing the appropriate button. The S2 stimulus of the spatial bisection corresponded to the S stimulus of the spatial localization (i.e., they were identical AV stimuli reproduced either at −2.3° or +2.3° in space), thus the two tasks presumably only differed in the kind of spatial representation that the AV stimuli elicited. Specifically, the bisection would elicit the construction of a complex spatial metric of S2 in relation to S1 and S3, as participants, after localizing the stimuli in space, had to link their spatial locations to each other into a single spatial layout. Instead, the localization would involve the spatial representation only of S. In principle, participants also in the bisection task could evaluate the position of only S2 and not of S2 in reference to S1 and S3, similarly to the localization task. Nevertheless, we are confident in excluding this possibility as a study using the same bisection task but with S1 and S3 positions that varied across trials (Aggius‐Vella et al., 2020) showed similar behavioral results to other studies with paradigms using fixed S1 and S3 (Gori et al., 2014, 2018; graphical evidence is reported in Figure S1). This observation suggests that, in the spatial bisection, participants refer S2 position to S1 and S3, thus that S2 of the spatial bisection and S of the spatial localization were presumably processed differently.

The two tasks were performed in two separate blocks, counterbalanced across subjects. Each block (i.e., each task) consisted of 240 trials and 15 catch trials (in which S2 and S were delivered at 0° and at 0 ms). The experimenter, together with the electrooculogram (EOG) signal, monitored participants to ensure they maintained a stable head position throughout the experiment.

### 2.3 Electroencephalography (EEG) data collection and pre‐processing

Electroencephalography (EEG) and EOG were recorded with 64 active electrodes using the Biosemi ActiveTwo EEG System. For the EOG recordings, two electrodes were positioned at the left and right outer canthi to check horizontal ocular movements.

Preamplifiers in each electrode reduced noise between the electrode and the amplification/digitization system electrode and the amplification/digitization system, allowing high electrode impedances. We kept electrode offsets under 35 mV. We applied a first‐order analog anti‐aliasing filter with a half‐power cutoff at 3.6 kHz and then down‐sampled data at 512 Hz (acquisition at 2048 Hz, with a decimation factor of 1/4) with pass‐band from direct current (DC) to 134 Hz. We referenced the EEG recording to a common mode sense active electrode and a driven right leg passive electrode. These play the role of the ground electrodes used in conventional systems.

EEG was filtered between 0.1 and 100 Hz. We applied the artifact subspace reconstruction (ASR) method, available as a plug‐in for EEGLAB software (Delorme & Makeig, 2004), to remove transient stereotypical (e.g., eye blinks) and non‐stereotypical (e.g., movement or muscle bursts) high‐amplitude artifacts. ASR uses a sliding window technique, which decomposes each window of EEG data via principal component analysis, allowing statistical comparison with data from a clean baseline EEG recording. Within each sliding window, the ASR algorithm identifies principal subspaces, which significantly deviate from the baseline and then reconstructs these subspaces using a mixing matrix computed from the baseline EEG recording. Our study selected a 500 ms sliding window and a threshold of three standard deviations to identify corrupted subspaces. Additionally, we removed channels that posed an inferior correlation with other channels (more than 0.85), or those with line noise relative to its signal presenting more than four standard deviations based on the total channel population. Finally, whenever the fraction of contaminated channels exceeded the threshold of 0.25, we removed time windows. We further cleaned EEG data using independent component analysis with two EEGLAB toolboxes, namely SASICA (Chaumon et al., 2015) and IC_MARC (Frølich et al., 2015). We kept all parameters at their default. We rejected components following criteria reported in the corresponding validation papers that chose rejection based on abnormal topographies and/or spectra. We referenced data to the average of the left and right mastoids (TP7, TP8 electrodes), to be consistent with previous studies using similar paradigm and analysis (Amadeo, Campus, et al., 2019; Amadeo, Störmer, et al., 2019; Campus et al., 2017, 2019; Gori et al., 2023).

### 2.4 Behavioral and sensor‐level analysis

We computed behavioral performance as the percentage of correct responses for each task. To consider possible different requirements of the two tasks, we also calculated task difficulty as the difference between the spatial localization and the spatial bisection behavioral performance.

The ERPs analysis focused separately on the neural responses to S2 for the spatial bisection task and to S for the spatial localization tasks. We focused on the second stimulus (S2) of the spatial bisection as it was physically identical to S of the spatial localization task (from which it differed only in relation to the experimental question). Moreover, S2 represents the starting point for constructing a metric spatial representation in the bisection task and previous studies demonstrated specific early occipital activation related to its processing (Campus et al., 2017, 2019; Gori et al., 2023). We obtained the ERPs by averaging EEG data synchronously with the S2 and S onsets. We considered as baseline a time window of 200 ms before S1 onset for the spatial bisection and 200 ms before S onset for the spatial localization. For each participant, the study required a minimum of 100 trials per block per ERP. The total number of trials for each ERP was equal to 3652 for bisection and 3627 for localization task, respectively and approximately corresponding to (mean ± SD) 214.82 ± 3.71 and 213.35 ± 4.46 per participant.

The analysis focused on electrodes related to visual processing (O1, O2 in occipital areas), given the task‐specific neural responses to spatial tasks that past studies revealed at these sites (Campus et al., 2017, 2019; Gori et al., 2023). We computed the mean ERP amplitude of two time windows after S2 and S onsets: between 50 and 90 ms, and between 110 and 160 ms. We chose these time windows to explore some main ERP processing stages of occipital areas (Di Russo et al., 2002; Hillyard & Anllo‐Vento, 1998). Specifically, we considered one time window corresponding to the visual C1 component (50–90 ms) which past studies indicated as involved in an early contralateral occipital response to spatialized sounds and lights (Campus et al., 2017, 2019; Gori et al., 2023); one time window encompassing the visual P100 (80–130 ms) and P140 (110–160 ms) components, which are associated with low‐level mechanisms of visual spatial attention (Di Russo et al., 2002). For the considered time windows, we collapsed average ERP waveforms across conditions (i.e., [a] S2 or S from −2.3° and [b] S2 or S from +2.3°; Figure 1) and hemispheres of recording so as to obtain ERPs recorded on the contralateral and the ipsilateral hemisphere with respect to stimulus position in space. We therefore calculated lateralized ERP responses as the difference between the contralateral and ipsilateral ERP recordings. By performing *t*‐test, we compared the cortical activity between the spatial bisection and the spatial localization tasks within the two selected time windows. We kept separate the statistical comparisons of the considered time windows as they refer to neurophysiological mechanisms not directly comparable to each other. We performed a similar sensor level analysis on central (C1 and C2 electrodes) and temporal areas (T7 and T8 electrodes) in the two time windows. In addition, to consider also task difficulty, for each time window we performed an analysis of covariance (ANCOVA) with the mean ERP occipital responses to S2 and S as dependent variable, *Task* as within factor and *Task difficulty* as covariate. Finally, to check for possible effects of the first stimulus on the second one, we computed ERP scalp topographies splitting between when S1 and S2 in the spatial bisection were separated by 500 ms or by 1000 ms.

### 2.5 Source level analysis

In our study, we employed distributed source analysis with Brainstorm software (Tadel et al., 2011). As in previous studies (Amadeo et al., 2020; Campus et al., 2017, 2019; Gori et al., 2020b, 2023), this provided evidence that the components we observed over the occipital scalp involved generators in the visual areas.

We re‐referenced data to the common average. We used a standard 1 mm resolution template of the Montreal Neurological Institute (nonlinear average of 152 subjects, processed with FreeSurfer 5.3 ICBM152, Fonov et al., 2009). As individual Magnetic Resonance Imaging scans were unavailable, to avoid misleading over‐interpretation, dipole orientations were not fixed to the cortex surface but rather free to assume whichever (unconstrained) orientation. We performed the EEG forward modeling using a three‐layer (head, outer, and inner skull) symmetric boundary element model (BEM) generated with OpenMEEG86. A diagonal noise covariance matrix was computed for each participant, using the pre‐stimulus interval to estimate sensor variance. We estimated source intensities using the sLORETA approach (Gramfort et al., 2011)—a technique that has been robust to noise in EEG recordings and head model approximations. We kept Brainstorm's default parameter settings for both source reconstruction and BEM creation.

The study separately averaged source activation for each subject and condition within the two selected time windows (50–90 and 110–160 ms) after S2 and S onsets. Subsequently, the norm of the vectorial sum of the three orientations at each vertex was estimated. Finally, paired *t*‐tests investigated pairwise comparisons corrected for multiple comparisons with the false discovery rate method, using *p* = .00001 as a threshold. We verified the modulation of the occipital activation during the spatial bisection and the spatial localization tasks. We did this by comparing the neural response between the two tasks, separately considering the stimulus positions in space (±2.3°).

## 3 RESULTS

### 3.1 Sensor level analysis

**FIGURE 2:**
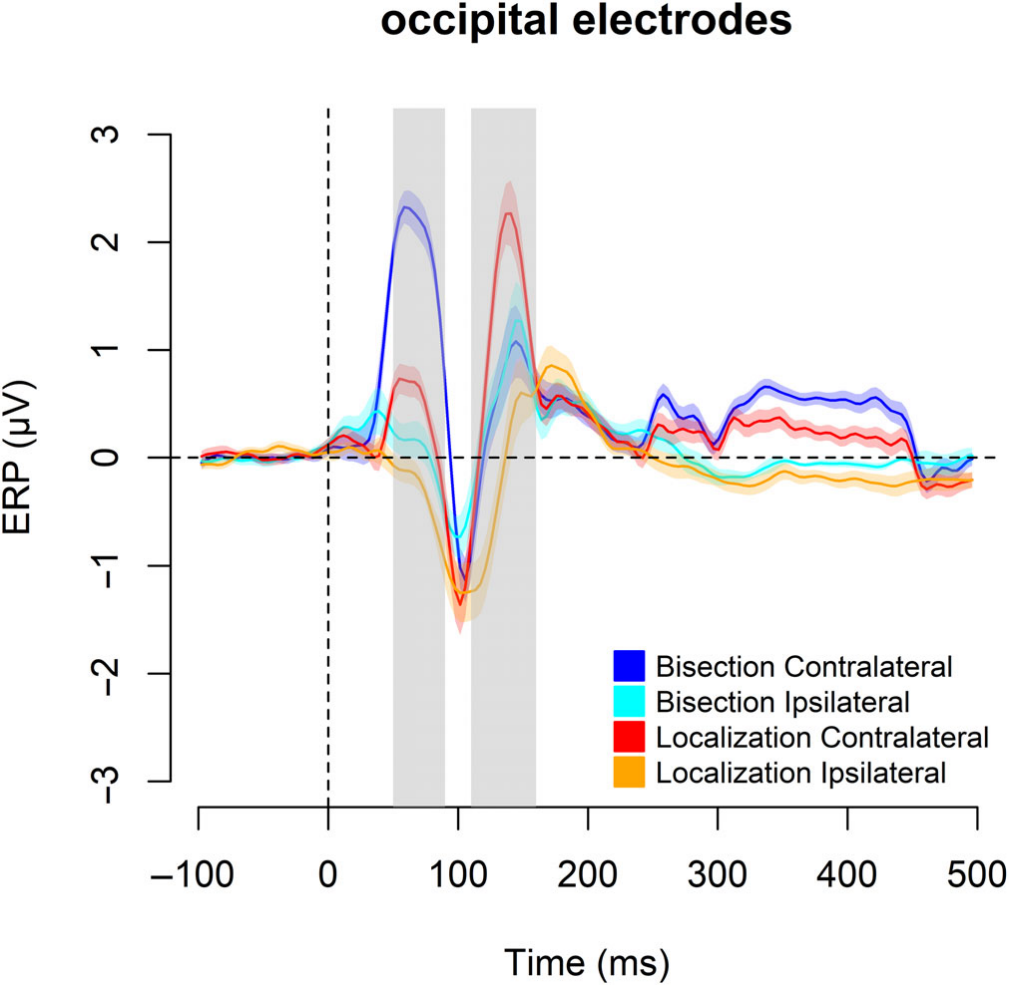
Contralateral and ipsilateral occipital event‐related potentials (ERPs) (mean ± standard error of means [SEM]) in respect to S2 during the spatial bisection task (blue and light blue curves) and in respect to S during the spatial localization task (red and orange curves). The gray‐shaded areas delimit the two time windows: 50–90 and 110–160 ms.

**FIGURE 3:**
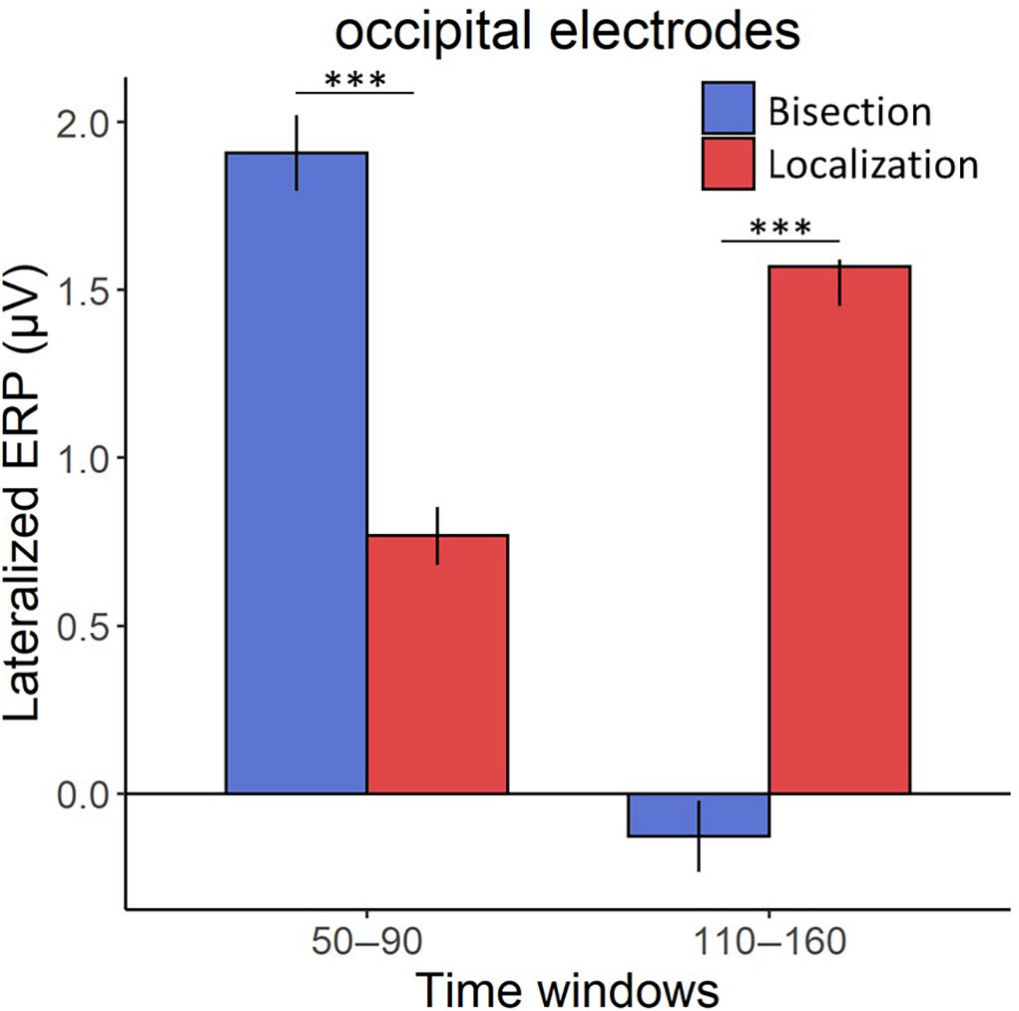
Lateralized mean event‐related potential (ERP) amplitude, calculated as the difference between the contralateral and ipsilateral ERP occipital responses in the 50–90 and 110–160 ms time windows separately, for the spatial bisection (blue bars) and spatial localization (red bars). Error bars indicate standard error of the mean (SEM). ***p < .001.

**FIGURE 4:**
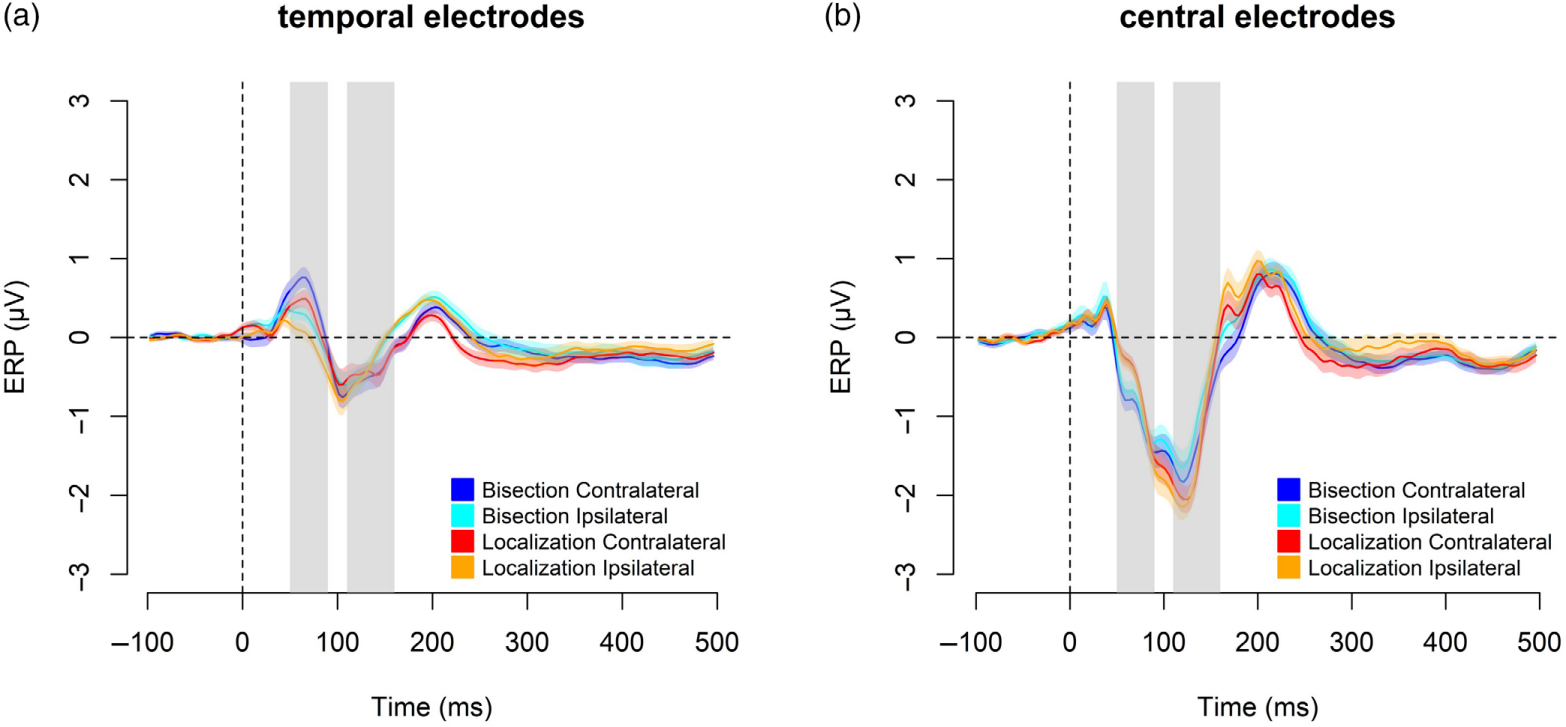
Contralateral and ipsilateral temporal (a) and central (b) event‐related potentials (ERPs) (mean ± SEM) in respect to S2 during the spatial bisection task (blue and light blue curves) and in respect to S during the spatial localization task (red and orange curves). In both panels, the gray‐shaded areas delimit the two time windows: 50–90 and 110–160 ms.

To compare the occipital response between the spatial bisection and the spatial localization task, we run hypothesis‐driven two‐tailed *t*‐tests on the mean ERP occipital responses to S2 and S. This is done within two separate time windows after stimuli onsets. In the 50–90 ms time window (Figure 2), results revealed an occipital positivity contralateral to the stimulus position in space (i.e., either −2.3° or +2.3°) during both spatial tasks. The occipital positivity resembled the C1 visual component, which may have originated from the primary visual cortex (Di Russo et al., 2002). This early occipital activation was significantly stronger during the spatial bisection task compared to during the spatial localization task (Figure 3; *t*[16] = 7.39, *p* < .001, Cohen's *d* = 1.79, 95% CI = [0.99, 2.59]). In the 110–160 ms time window, the spatial bisection task evoked a bilateral occipital response resembling a later phase of the P1 (i.e., P140, peak latency of 146 ms), while the spatial localization task elicited an occipital activation contralateral to the S position in space, which recalls an earlier phase of the P1 component with a peak latency of 110 ms (Figure 2). In addition, the neural activation in this time window was significantly greater during the spatial localization than during the spatial bisection (Figure 3; *t*[16] = −19.85, *p* < .001, Cohen's *d* = −4.81, 95% CI = [−6.56, −3.06]). Overall, results on the occipital sites suggested that later ERP components (110–160 ms post‐stimulus) were more pronounced for the identification of a single point in space (as in the spatial localization) than with the metric representation of the space (as in the bisection) which conversely was associated with a stronger earlier occipital activation (50–90 ms post‐stimulus). Two tailed *t*‐tests on the mean ERP at central (i.e., C1 and C2 electrodes) and temporal locations (i.e., T7 and T8 electrodes) within the two time windows (considered as reflecting more specifically the auditory aspect of stimuli processing) did not show significant differences between the two tasks (Figure 4; temporal sites: 50–90 ms: *t*[16] = 0.04, *p* = .964, Cohen's *d* = 0.01, 95% CI = [−0.48, 0.50]; 110–160 ms: *t*[16] = −0.38, *p* = .705, Cohen's *d* = −0.09, 95% CI = [−0.58, 0.40]. Central sites: 50–90 ms: *t*[16] = −1.87, *p* = .079, Cohen's *d* = −0.45, 95% CI = [−0.97, 0.06]; 110–160 ms: *t*[16] = 1.66, *p* = .115, Cohen's *d* = 0.40, 95% CI = [−0.11, 0.91]). The observation that cortical activity was not influenced by the kind of spatial task in temporal and central regions, but it was in the occipital areas, suggested that modulation by the nature of the spatial processing is specific for the occipital activation, likely because these sites play a crucial role in the spatial perception.

**FIGURE 5:**
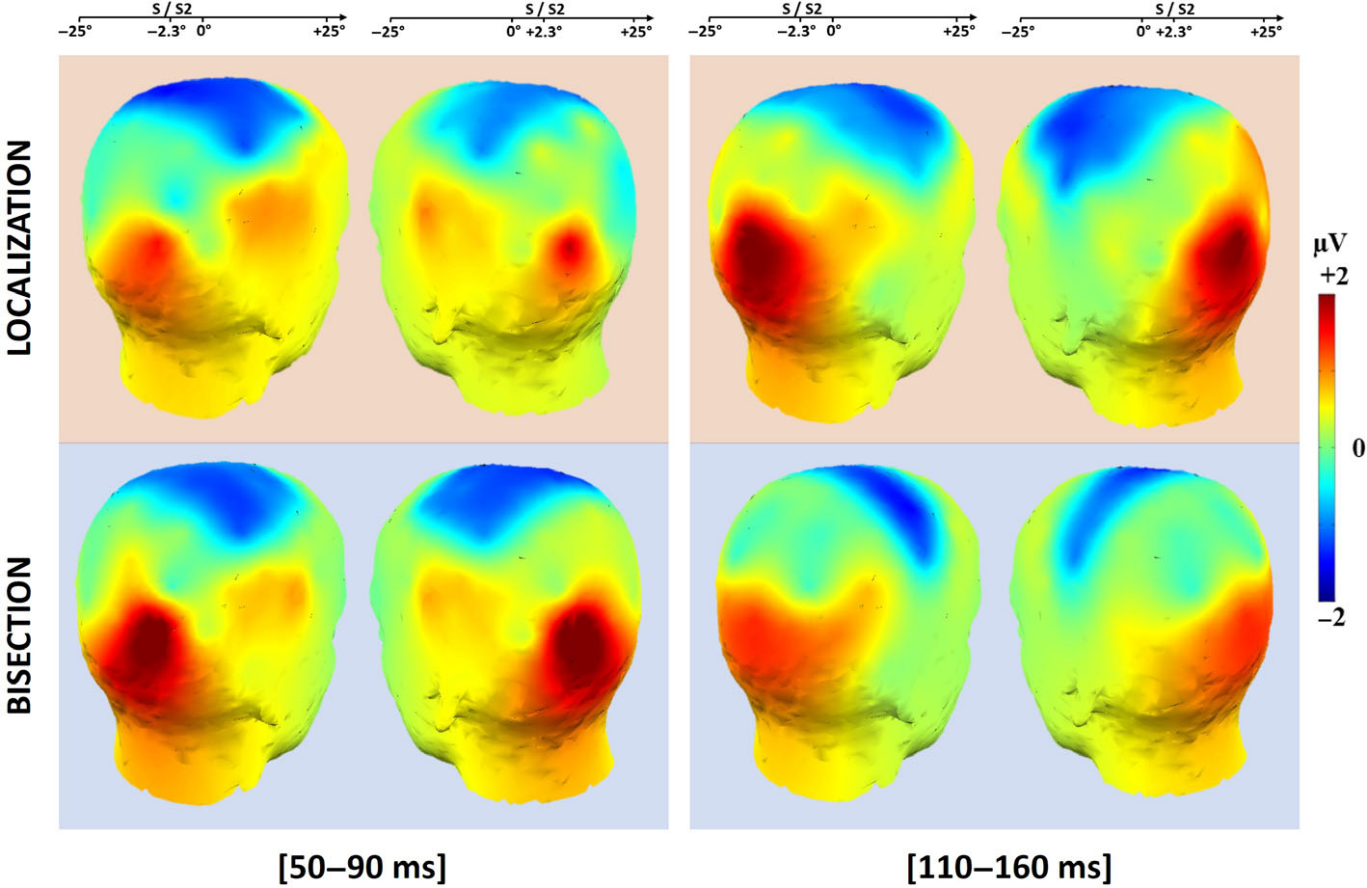
Scalp maps of the mean event‐related potential (ERP) amplitude in the 50–90 ms (left column) and 110–160 ms (right column) time windows after S2 and S onsets, for the spatial bisection (lower row) and the spatial localization (upper row), respectively. Above each column the conditions to which the scalp topographies refer are reported.

In Figure 5, the scalp topographies of the mean ERPs in the 50–90 ms and 110–160 ms time windows show these results. In the earliest time window (50–90 ms), the contralateral occipital response was more prominent during the spatial bisection than during the spatial localization. In the later time window (110–160 ms), the results were reversed with greater lateralized occipital activation for the spatial localization task than for the spatial bisection. Finally, the scalp maps of the mean ERP response divided between (i) when S1 preceded S2 of 500 ms and (ii) when S1 preceded S2 of 1000 ms (Figures S3 and S4) showed that the neural response between these two conditions did not significantly differ, for the two time windows. This observation suggests that the presentation of S1 before S2 should not influence the cortical response associated with S2 in the spatial bisection.

### 3.2 Source level analysis

**FIGURE 6:**
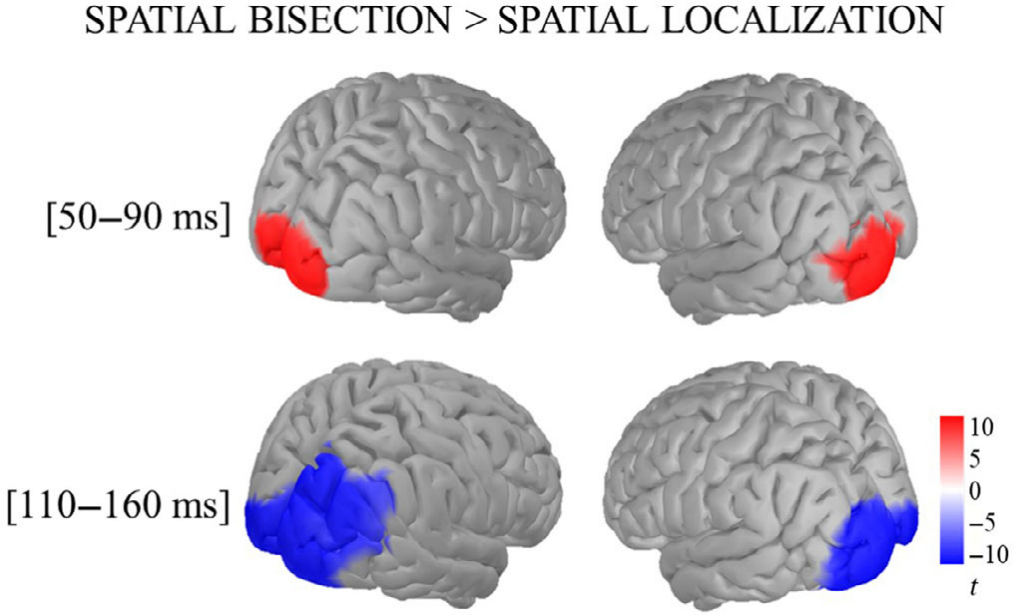
Maps of the paired two‐tailed *t*‐tests run on the average source activity in the 50–90 ms (top row) and the 110–160 ms (bottom row) time windows: reddish and bluish colors indicate stronger activation during the spatial bisection and the spatial localization task, respectively. The significance of the difference (i.e., the magnitude of *t*) is represented by color intensity. Left and right columns show the conditions in which S/S2 were reproduced at −2.3° or +2.3°, respectively.

Despite the low spatial resolution of the EEG technique, we provided further evidence of the cortical generators of the ERP components during the two spatial tasks, by performing statistical comparisons in a source‐level analysis (Figure 6). In the analysis, we considered the neural response at S2 and S for the spatial bisection and the spatial localization, respectively. In the 50–90 ms time window, a paired two‐tailed *t*‐test indicated significantly greater recruitment of contralateral occipital areas during the spatial bisection than during the spatial localization task. On the contrary, within the 110–160 ms time window, the contralateral activation was more pronounced during the spatial localization, in similar cortical areas than in the earlier time window. These posterior cortical activations likely involved visual areas for both spatial tasks.

### 3.3 Behavioral performance

Participants correctly localized significantly more AV stimuli in the spatial localization task than in the spatial bisection task (mean ± SD percentage of localization: 98.18 ± 2.17; mean ± SD percentage of bisection: 91.76 ± 7.93; *t*[16] = 4.07, *p* = <.001, Cohen's *d* = −0.98, 95% CI = [−1.59, −0.38]). To consider also the task difficulty in the neural response to the tasks, we performed an ANCOVA with *Task difficulty* as a covariate. The ANCOVA revealed that the main effects of *Task* remained under control for the task difficulty in the occipital sites for the time windows considered (50–90 ms: *Task*: *F*(1,15) = 53.61, *p* < .001; *Task difficulty: F*(1,15) = 4.67, *p* = .047; *Task* × *Task difficulty*: *F*(1,15) = 0.68, *p* = .42. 110–160 ms: *Task*: *F*(1,15) = 377.94, *p* < .001; *Task difficulty*: *F*(1,15) = 14.74, *p* = .001; *Task* × *Task difficulty*: *F*(1,15) = 0.34, *p* = .566).

## 4 DISCUSSION

Across different species, space is a fundamental dimension upon which perception and behavior are organized. Dealing with spatial information can be a challenging task as it requires one to construct a uniform spatial layout starting from different sensory systems and different spatial representations of the environmental inputs. External stimuli can be localized in coordinates that account for the spatial relations among the inputs, or for coordinates that refer only to the position in space of the perceiving subject. At the cortical level, studies have revealed evidence of a spatial representation system in the human brain, where neural responses occurred at different levels of sensory processing and within several cortical areas (Gori et al., 2020a, 2023; Molholm et al., 2002; Retsa et al., 2020). However, less is known about the modulation of this neural activity in response to different kinds of spatial processing, as the spatial representations of different nature have never been compared at the cortical level. To fill this gap, in the present study we investigated the recruitment of occipital areas when participants responded to multisensory stimuli embedded in two tasks relying on different spatial processing. We required participants to process an identical AV stimulus: (i) in a spatial bisection task by estimating the relative position of the AV stimulus within a sequence of three spatialized AV stimuli and (ii) in a spatial localization task by localizing the position of the AV stimulus itself. Participants' behavioral and EEG data were recorded during both spatial tasks.

Our results showed that early occipital components were more robust during a spatial representation that required a metric definition among multiple stimuli, while later occipital components during a spatial localization of a single position in space. Specifically, an early occipital activation (50–90 ms) contralateral to the AV stimulus location was stronger when participants encoded the stimuli in the spatial bisection task, compared to the spatial localization task. Previous studies observed similar occipital responses in an early post‐stimulus onset interval (which was defined as early‐latency multisensory interaction [eMSI]; reviewed in De Meo et al., 2015) in response to multisensory spatialized stimuli (Molholm et al., 2002) and to multisensory inputs within a spatial bisection task (Gori et al., 2023). However, two spatial tasks were directly compared to each other only in the present study. This comparison showed a modulation of the occipital activity within the eMSI by the spatial features involved in the task, with a stronger activation during the spatial bisection than the spatial localization. In a later time window (110–160 ms), we revealed a neural modulation as well. However, in this case, the spatial localization task elicited a greater and more lateralized occipital response than the spatial bisection task. Previous studies revealed similar patterns of activation in this time range by investigating the cortical responses associated with multisensory spatial tasks (Gori et al., 2023; Molholm et al., 2002).

Research indicated that a subject's goals influence the cortical responses to multisensory stimuli (Talsma et al., 2010; ten Oever et al., 2016), with a circuit‐based description of sensory mechanisms that accounts, for example, for the complexity of the factors involved in this processing (reviewed in Murray, Lewkowicz, et al., 2016). A similar pattern may emerge for the ability to represent space, with modulation of occipital areas' activity in response to multimodal inputs that depends on the subject's goal, in this case on the kind of spatial representation a person builds. The fact that we revealed such modulation specifically within the occipital areas and not within other regions of interest of the temporal and central scalp is supported by the crucial role of visual areas in the spatial representation (Gori et al., 2023). The reason why the neural modulation occurs in this way, that is, with an earlier activation for a task of spatial metric construction and a later response for a localization task of a single point, remains a matter of interpretation. Nevertheless, we speculate that the cross‐sensory calibration that occurs between the visual modality and the other sensory systems for the development of some spatial skills may have a role in this schema (Gori, 2015). A simple task such as localization may not require cross‐sensory visual calibration and, consequentially, it may make recruiting early occipital components for processing this type of spatial information less substantial. In line with this, research indicates that localization skills do not require visual experience to properly develop (Battal et al., 2020; Collignon et al., 2009), as both sighted and blind infants showed similar reaction times and accuracy in an audio localization task (Gori et al., 2021). On the contrary, a more complex task, such as bisection, may benefit more from visual calibration and, consequently, from the support of early cortical response (Murray, Lewkowicz, et al., 2016; Van Atteveldt et al., 2014). In this regard, it has been suggested that “the functional implications of such ‘early’ multisensory influences are likely to be substantial in that they suggest a previously unrealized level of interaction that could confer tremendous flexibility on sensory function (Van Atteveldt et al., 2014)” (Murray, Lewkowicz, et al., 2016), thus, for the bisection task an early neural computation may provide the flexibility needed for processing complex multisensory requests. The fact that blind individuals, who lack visual calibration of auditory and tactile spatial abilities, do not show this early activation and, consequentially, are unable to perform the spatial bisection task (Campus et al., 2019; Gori et al., 2020b; Tonelli et al., 2020) seems to support this speculation. Similarly, studies on the multisensory spatial abilities of children showed that, for a spatial bisection task, visual dominance exists before optimal multisensory integration mechanisms (Gori et al., 2008, 2012), while, for an AV spatial localization, multisensory capabilities develop before cross‐sensory calibration (Rohlf et al., 2020). Altogether, we speculate that the modulation of occipital activation revealed between the two spatial tasks may be due to a different role of visual calibration for processing different spatial information: more important for the bisection rather than for the localization task.

One limitation of the present study is the lack of unisensory (visual or auditory) conditions to compare with the multisensory (audio‐visual) condition of the present study. This aspect limits the claim that we measured phenomena of multisensory processing at the level of the occipital cortex, as we cannot exclude that participants were processing only the visual stimuli as they were the most relevant inputs for spatial computation. However, if we qualitatively compare our results with those of past studies exploiting similar tasks but with unimodal conditions (spatial bisection: visual stimuli: Amadeo et al., 2020; auditory stimuli: Campus et al., 2017, 2019; spatial detection: visual stimuli: Teder‐Sälejärvi et al., 2005; auditory stimuli: Amadeo, Störmer, et al., 2019) we observed the multisensory response of our study to be, in most cases, larger than the unisensory response of these previous studies (graphical evidence of the occipital response to unisensory spatial bisection and localization paradigms of past studies is reported in Figure S2). This qualitative observation suggests that our neural response was likely related to multisensory processing. However, future investigations directly comparing unisensory and multisensory spatial stimuli are still needed. Another limit is the presence of S1 in the spatial bisection but not in the spatial localization which may had a role in the cortical difference we revealed between the two tasks. However, (i) we did not find any significant difference between when S2 was reproduced 500 or 1000 ms after S1 (as also revealed in past studies using the spatial bisection; Amadeo, Campus, et al., 2019; Amadeo et al., 2020; Gori et al., 2020b), (ii) we did not find any significant difference between when S2 was reproduced at −2.3° or +2.3°, and (iii) attentional effects of S1 on S2 (e.g., pre‐cueing, temporal masking, or attentional blink effects) were discarded as the two stimuli were separated by a large temporal distance (500 or 1000 ms). For all these reasons, we are confident in excluding an influence of S1 on S2 that was undetectable in the spatial localization due to the lack of S1 in this task. Finally, the spatial resolution of the EEG technique does not allow certainty in considering the occipital activation we revealed as involving visual areas.

To conclude, the present study demonstrated a different recruitment of the occipital cortical network based on the kind of spatial processing elicited by environmental stimuli. These results may have important implications for future works, such as in exploring the neural processing associated with different spatial skills and investigating cortical plasticity in blindness.

## CONFLICT OF INTEREST STATEMENT

The authors declare that there is no conflict of interest that could be perceived as prejudicing the impartiality of the research reported.

## Supporting information


**Data S1.** Supplementary Information.

## Data Availability

The dataset presented in this study can be found in online Zenodopublic repository at the following link: https://zenodo.org/record/8372651.
