# Supplementary material for: Task‐dependent spatial processing in the visual cortex
Source: Hum Brain Mapp. 2023 Oct 9;44(17):5972–81. doi: 10.1002/hbm.26489 (PMC10619374; doi:10.1002/hbm.26489)
Supplement: Supplementary file 1 — Data S1. Supplementary Information. [file HBM-44-5972-s001.docx]

**Supplementary information**

**Task-dependent spatial processing in the visual cortex**

*Bertonati G.^1,2^, Amadeo M.B.^1*^, Campus C.^1^ & Gori M.^1^*

^1^Unit for Visually Impaired People (U-VIP), Istituto Italiano di Tecnologia, Genoa, Italy

^2^Department of Informatics, Bioengineering, Robotics and Systems Engineering (DIBRIS), Università degli Studi di Genova, Genoa, Italy

*Corresponding author

**Contact details:**

Giorgia Bertonati: giorgia.bertonati@iit.it

Maria Bianca Amadeo: mariabianca.amadeo@iit.it

Claudio Campus: claudio.campus@iit.it

Monica Gori: monica.gori@iit.it


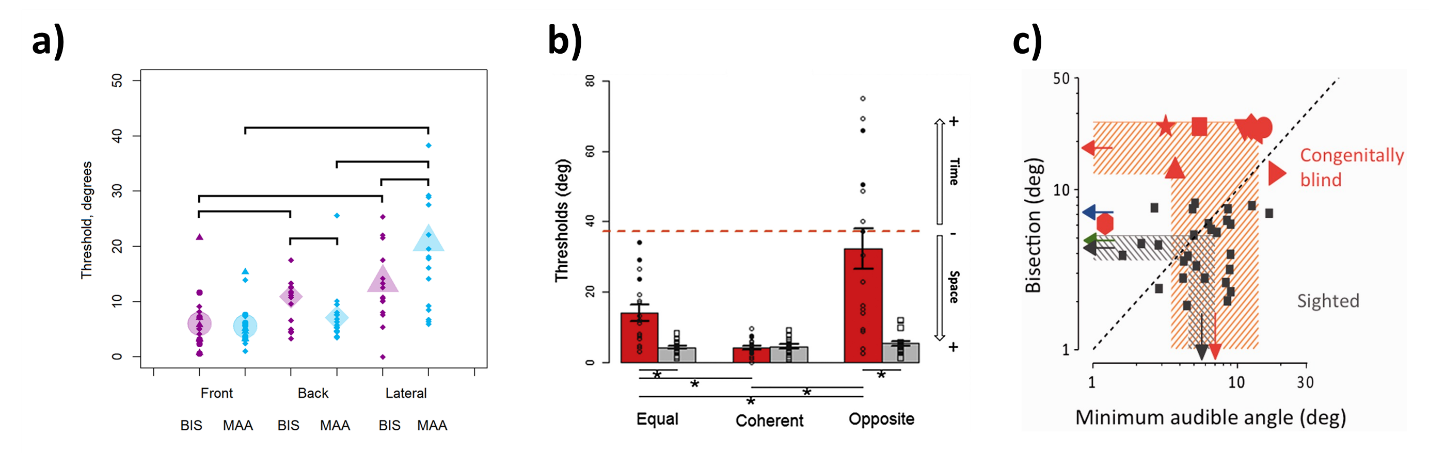


**Figure S1: behavioral results of previous studies using the spatial bisection task with fixed or variable S1 and S3 spatial positions:** a) Average thresholds in a minimum audible angle task (MAA) and in a spatial bisection task (BIS) for front (circle), back (rhombus) and lateral (triangle) stimuli positions for sighted adults, from *Aggius-Vella, E., Kolarik, A. J., Gori, M., Cirstea, S., Campus, C., Moore, B. C. J., & Pardhan, S. (2020). Comparison of auditory spatial bisection and minimum audible angle in front, lateral, and back space. Scientific Reports, 10(1), 1–9*. In this study, S1 and S3 of the spatial bisection task varied in their spatial positions and could be randomly reproduced at ±22°, ±17.6°, or ±13.2° in different trials. b) Average thresholds for a frontal spatial bisection task with equal, coherent, or opposite temporal and spatial features, for blind (red bars) and sighted (gray bars) participants, from *Gori, M., Amadeo, M. B., & Campus, C. (2018). Temporal Cues Influence Space Estimations in Visually Impaired Individuals. IScience, 6, 319–326*. In this study, S1 and S3 of the spatial bisection task were reproduced at -25° and +25°, respectively, in every single trial. c) Individual data (dots) and average thresholds (arrows) in a frontal spatial bisection task and a minimal audible angle task for sighted (red symbols and shaded areas) and blind participants (grey symbols and shaded areas), from *Gori, M., Sandini, G., Martinoli, C., & Burr, D. C. (2014). Impairment of auditory spatial localization in congenitally blind human subjects. Brain, 137(1), 288–293.* In this study, S1 and S3 of the spatial bisection task were reproduced at -25° and +25°, respectively, in every single trial.

Figures were reproduced and adapted with the permission of the above-mentioned papers’ authors.

**Figure S2:
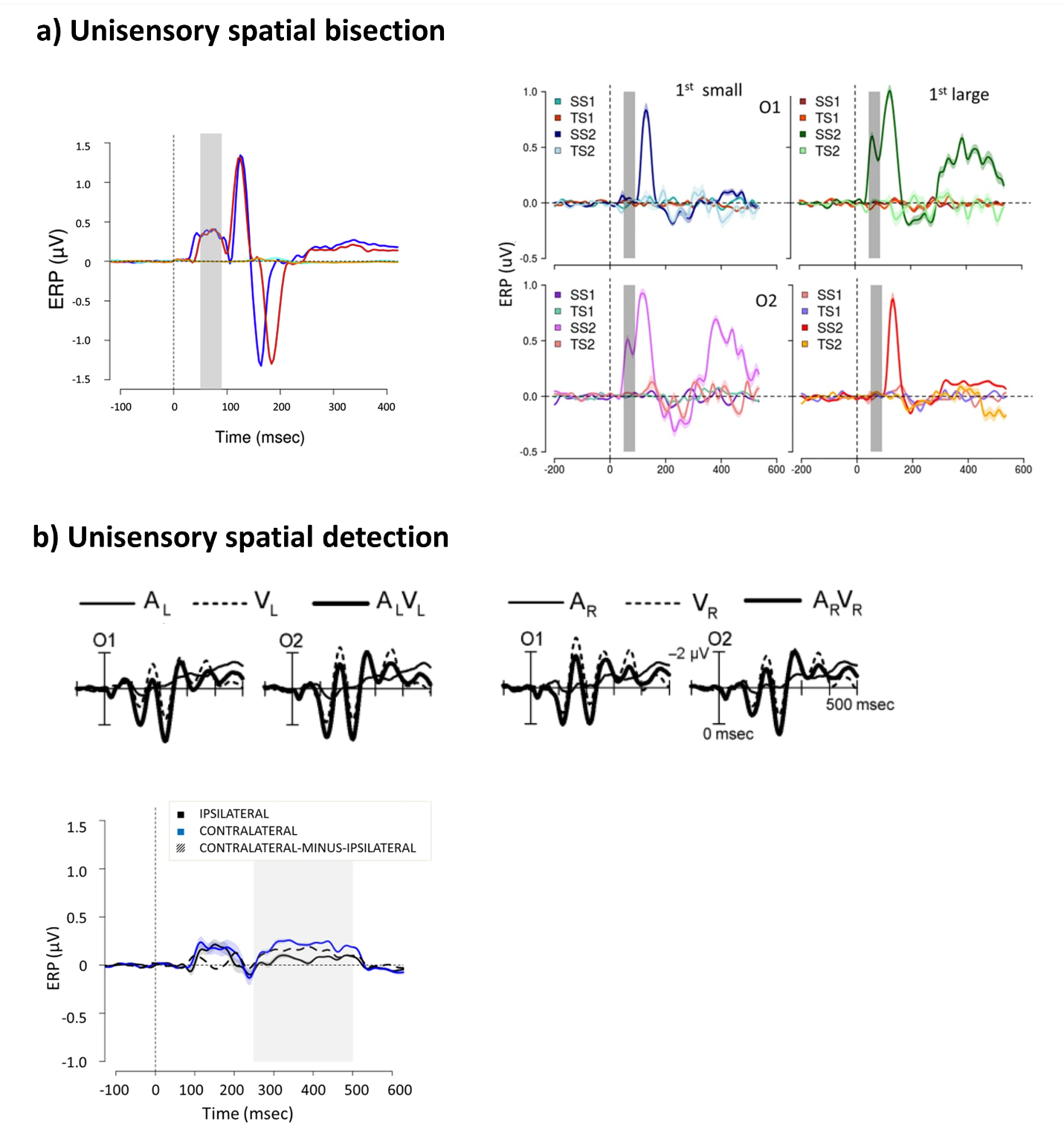
occipital activation during unisensory (auditory or visual) spatial bisection and spatial detection tasks.** a) Unisensory spatial bisection: Left panel: occipital activation during a visual spatial bisection task (red curve; from *Amadeo, M. B., Campus, C., & Gori, M. (2020). Visual representations of time elicit early responses in human temporal cortex. NeuroImage, 217(January), 116912*). Right panel: occipital activation during an auditory spatial bisection task (blue, green, pink, and red curves; from *Campus, C., Sandini, G., Concetta Morrone, M., & Gori, M. (2017). Spatial localization of sound elicits early responses from occipital visual cortex in humans. Scientific Reports, 7(1), 1–12*). b) Unisensory spatial detection: Upper panel: occipital activation during a visual spatial detection task (dashed curves; from *Teder-Sälejärvi, W. A., Di Russo, F., McDonald, J. J., & Hillyard, S. A. (2005). Effects of spatial congruity on audio-visual multimodal integration. Journal of Cognitive Neuroscience, 17(9), 1396–1409*). Lower panel: occipital activation during an auditory spatial detection task (from *Amadeo, M. B., Störmer, V. S., Campus, C., & Gori, M. (2019). Peripheral sounds elicit stronger activity in contralateral occipital cortex in blind than sighted individuals. Scientific Reports, 9(1), 1–10*). See Figure 2 for comparing these data with results in the audiovisual spatial bisection and spatial localization tasks of the present study.

Figures were reproduced and adapted with the permission of the above-mentioned papers’ authors.


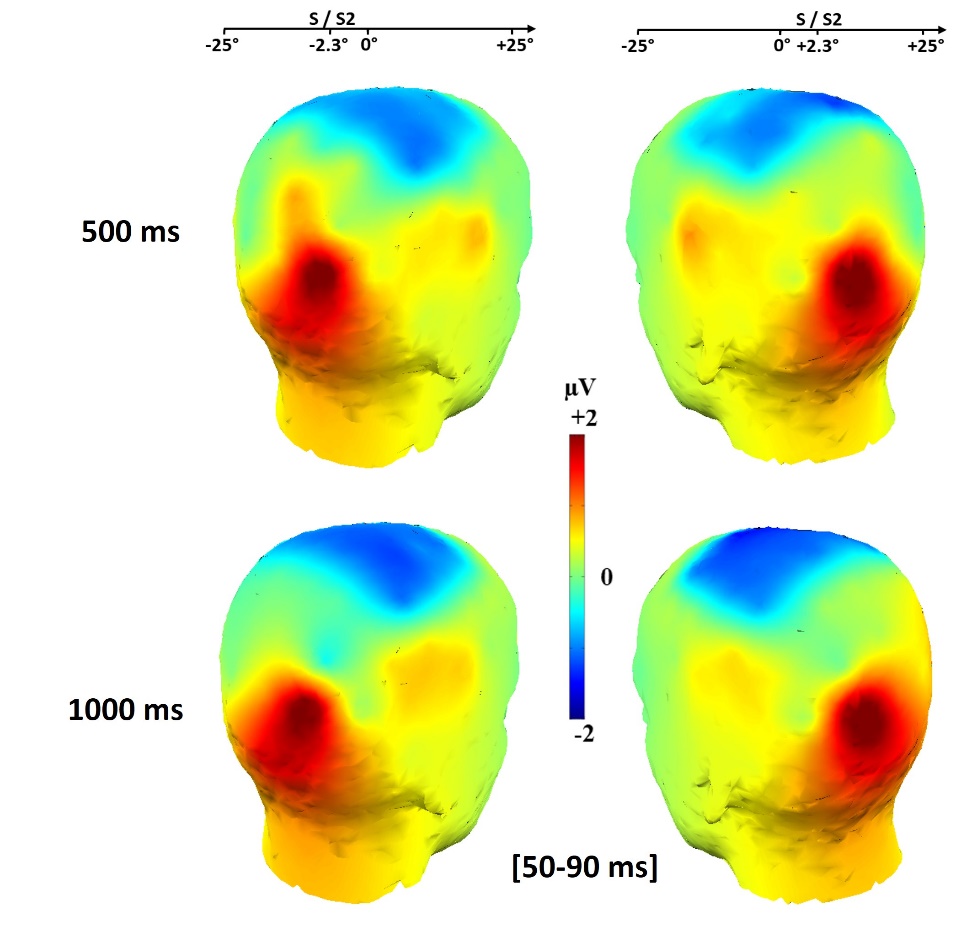


**Figure S3: Scalp maps of the mean ERP amplitude in the 50–90 ms time windows after S2 of the spatial bisection.** Left and right columns show the conditions in which S2 was reproduced at -2.3° or + 2.3°, respectively. The average neural response of when S2 was reproduced 500 ms after S1 (upper row) was similar to the average neural response of when S2 was presented 1000 ms after S1 (lower row).


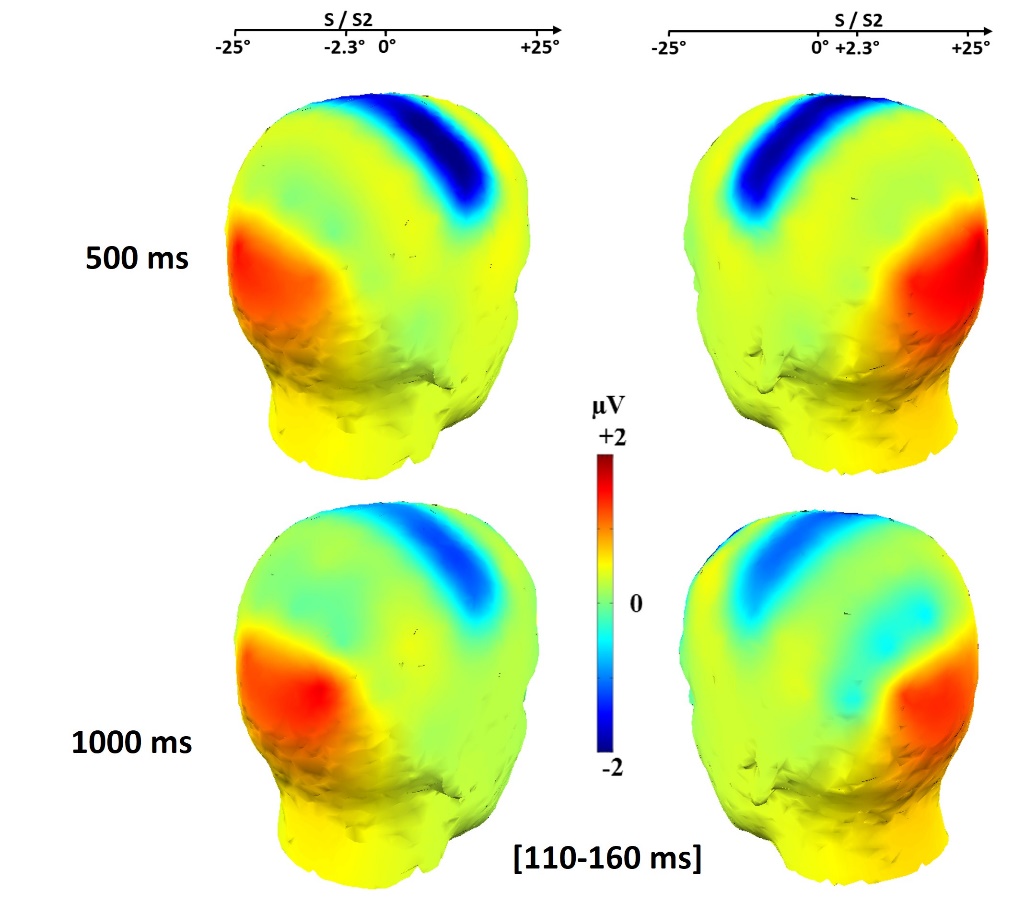


**Figure S4: Scalp maps of the mean ERP amplitude in the 110-160 ms time windows after S2 of the spatial bisection.** Left and right columns show the conditions in which S2 was reproduced at -2.3° or + 2.3°, respectively. The average neural response of when S2 was reproduced 500 ms after S1 (upper row) was similar to the average neural response of when S2 was presented 1000 ms after S1 (lower row).
